# Organohydrogel Based Electronic Skin Reinforced by Dual‐Mode Conduction and Hierarchical Collagen Fibers Skeleton

**DOI:** 10.1002/advs.202412934

**Published:** 2024-12-16

**Authors:** Ruyue Guo, Yan Bao, Xi Zheng, Jie Chen, Wenbo Zhang, Chao Liu, Jianzhong Ma

**Affiliations:** ^1^ College of Bioresources Chemical and Materials Engineering Shaanxi University of Science and Technology Xi'an 710021 P. R. China

**Keywords:** collagen fiber skeleton, conductive organohydrogel, dual‐mode conduction, electronic skin, flexible sensor

## Abstract

Collagen fiber skeleton from animal skin is an ideal substrate for electronic skin (e‐skin). However, the interface mismatch between conductive materials and skeleton and the monotonicity of conductive network still hinder its creation. Herein, a novel collagen fiber‐based e‐skin with dual‐mode conduction of NaCl and conductive spheres (IECS) is accomplished by loading organohydrogel into the skeleton via “permeation and self‐assembly”. The resulting interpenetrating network produces a 3D continuous, conductive pathway and strong interface interaction with high‐density hydrogen bonding, thus exhibiting excellent strength (24.5 MPa), conductivity (14.82 S m^−1^), sensing performance (sensitivity of 16.64), and environmental stability. The physical structure (3D skeleton, interpenetrating network) and chemical interaction (interface interaction, salting‐out) achieve energy dissipation. Meanwhile, the sensitivity is enhanced by dual‐mode conduction, conductive sphere array, and deformation amplification induced by collagen fibers. Additionally, the strong bonding ability between glycerin and collagen fibers with water molecules provides anti‐freezing and moisture‐retention characteristics. Thus, the strategic synergy of compositional and structural design makes IECS a promising force‐sensing part of piezoresistive sensor for human movement, pulse frequency, cipher transmission, and pressure distribution. In short, IECS presents a multifunctional platform for the invention of high‐performance e‐skin with on‐demand property, which offers great application potential in wearable electronics.

## Graphical abstract

This work creates a novel collagen fiber‐based e‐skin with dual‐mode conduction by loading organohydrogel into the collagen fiber skeleton via ″permeation and self‐assembly″, which simultaneously realizes enhanced excellent strength, conductivity, sensing performance, and environmental stability. It provides a multifunctional platform for the invention of high‐performance e‐skin with on‐demand property and offers great application potential in wearable electronics.

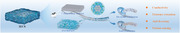

## 1 Introduction

The growing need for people to take responsibility for their own medical problems is an advancement in the concept of health building.^[^ ^1^, ^2^ ^]^ The advanced flexible sensor has accomplished significant progress and is currently gaining widespread application in multiple fields of bio‐sensing, electronic skin (e‐skin), health monitoring and soft robots. It operates by mimicking the biological sensing mechanism of nerves, replicating the topological structure and the tactile sensing function similar to human skin.^[^ ^3^ ^–^ ^5^ ^]^

The design and development of e‐skin has utilized an extensive selection of renewable resources and their synthetic counterparts (such as animal skin, silk, paper, etc) over the last several years.^[^ ^6^, ^7^ ^]^ Among them, collagen fiber is derived from natural animal skin, whose basic network is a 3D hierarchical structure. It has proven to be advantageous in biomedical materials, food, cosmetic, and traditional leather industries due to its high strength, good flexibility, bio‐compatibility, and simplicity in carrying other materials. Definitely, collagen fiber is a novel and fascinating substrate for the fabrication of e‐skin.^[^ ^8^ ^]^ Therefore, the goal of this work is to create an innovative type of collagen fiber‐based e‐skin (also known as skin‐based e‐skin) by preserving the intact, 3D, and cross‐linked network of collagen fiber in natural skin as the skeleton structure and incorporating other molecules as the sensing material. In this construction strategy, collagen fiber serves as a load‐bearing and dissipative scaffold. By facilitating the slip and deformation of collagen fiber skeleton with a hierarchical structure, it dissipates external mechanical energy and provides the corresponding e‐skin with high mechanical strength and toughness. As well, it is also simple, quick, and scalable for creating collagen fiber‐based e‐skin utilizing natural animal skin.

The primary purpose of collagen fiber‐based e‐skin is to recognize external signals and translate them into basic electrical signals.^[^ ^9^ ^]^ In this regard, adequate conductivity is an essential prerequisite for sensing function. The present work on collagen fiber‐based e‐skin predominantly emphasizes inorganic conductive materials and conductive polymer materials. On the one hand, collagen fiber‐based e‐skin with outstanding conductive properties is primarily generated by vacuum filtration relying on the size advantage of inorganic conductive materials. However, since inorganic material is brittle, it is challenging to integrate it into a flexible collagen fiber skeleton throughout a broad area. Meanwhile, the inorganic material eventually falls off because of the weak interaction and the interface mismatch effect with the collagen fiber skeleton, resulting in the restricted sensing stability of collagen fiber‐based e‐skin.^[^ ^10^, ^11^ ^]^ On the other hand, collagen fiber‐based e‐skin is created by the reactive activation of conductive polymer precursor through in situ polymerization. The corresponding e‐skin possesses good stability thanks to the flexibility of conductive polymer and it is in situ production between collagen fiber skeleton, meeting the demands of large‐area manufacturing of flexible electronics. Unfortunately, this process depends on the penetration of conductive polymer precursor within the collagen fiber skeleton and the conditions of polymerization, which frequently ends in a small amount of conductive polymer and poor conductivity of collagen fiber‐based e‐skin.^[^ ^12^, ^13^ ^]^ The aforementioned evidence indicates that the research system of collagen fiber‐based e‐skin has been enriched by various designs and preparations, which have established the foundation for its generation and utilization. But there nevertheless remain some shortcomings. In addition, collagen fiber skeleton derived from animal skin is not resistant to high temperatures, strong acids, and strong alkalis, limiting its combination with sensing materials. Therefore, it remains a challenge to construct collagen fiber‐based e‐skin that simultaneously delivers good conductivity, sensing ability, mechanical property, and versatility, which requires the exploration of new approaches and better materials.

Given that conductive hydrogel is a 3D soft material with high elasticity and good flexibility, many researchers have been particularly interested in it.^[^ ^14^, ^15^ ^]^ The utilization of conductive hydrogel as the sensing material offers another fascinating option for the construction of collagen fiber‐based e‐skin.^[^ ^16^, ^17^, ^18^ ^]^ Specifically, the primary challenges of this strategy include the uniform distribution of conductive hydrogel solution through collagen fiber skeleton in the flow state and the in situ encapsulation of conductive hydrogel by transforming it from a flow to a solid state.^[^ ^19^, ^20^, ^21^ ^]^ In current studies, the conductivity of hydrogel is primarily achieved through either ionic conduction or electronic conduction. The current conduction process of ion‐conducting hydrogel is achieved by facilitating the migration of ions in ionic liquids or inorganic salts. The resistance decreases with stretching and is directly proportional to the degree of deformation. Therefore, the ion‐conducting hydrogel has lower sensitivity at small strains but a wider sensing range.^[^ ^22^ ^]^ Whereas, electron‐conducting hydrogel utilizes electrons and holes to transmit electrical signals. The percolation network generated by the contact of conductive materials determines its electrical behavior. Due to the complete separation of the percolation network under large strain, the electron‐conducting hydrogel has a limited working range but higher sensitivity under small strain.^[^ ^23^ ^]^ In addition, the mechanical strength, anti‐freezing at low temperatures, and moisturization for a long time of conductive hydrogel are also the main issues that need to be focused on.^[^ ^24^ ^]^

Based on the above background, a straightforward “permeation and self‐assembly” technique was employed to create a novel collagen fiber‐based e‐skin (IECS) with an interpenetrating network structure by introducing conductive materials, organohydrogel network, and anti‐freezing agent into collagen fiber skeleton. It overcame the drawbacks of the current collagen fiber‐based e‐skin. In addition, it enhanced the mechanical strength, anti‐freezing, and moisture‐retention performances of organohydrogel. The interpenetrating network structure between collagen fiber and organohydrogel was investigated to satisfy the requirements of synergistic deformation and continuous electrical signal transmission. As conductive media, the effects of polymethyl methacrylate@Ti_3_C_2_T_X_ (PMMA@MXene, denoted as PM) spheres and inorganic ions (Na^+^, Cl^−^) on the electrical and sensing properties of IECS were also studied systematically. In the meantime, IECS developed a dense hydrogen bonding structure upon the addition of glycerin, and its moisture‐retention, anti‐freezing, and self‐regenerating were also illustrated. It was worth noting that the effect of collagen fiber on the comprehensive properties of IECS was elucidated to confirm its necessity. The resulting IECS had excellent mechanical strength, conductivity, sensitivity, anti‐freezing properties, and moisture retention, which offered considerable advantages for precise acquisition of human movement, pulse frequency, cipher transmission, and pressure distribution. These advantages made IECS a promising candidate for wearable electronics, human‐machine interfaces, and artificial intelligence, providing a new platform for the realization of multifunctional flexible e‐skin.

## 2 Results and Discussion

### 2.1 Design Principle and Synthesis of IECS

**Figure 1:**
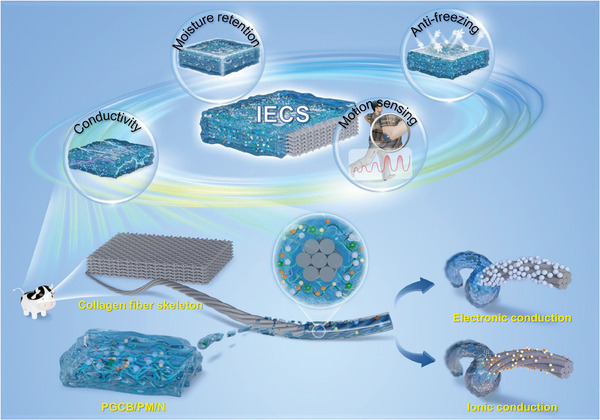
Schematic illustration of the preparation process of multi‐functional IECS.

Collagen fiber bundles with numerous binding sites and hierarchical structures, as the skeleton and carrier of IECS, offered an ideal structural foundation for the addition of functional materials. The final IECS with dual‐mode conduction, high mechanical strength, low‐temperature tolerance, and moisturization was constructed in this work by employing an innovative “permeation and self‐assembly” strategy based on stuffing and nano‐engineering, as illustrated in **Figure** **1**. The whole procedure was divided into four stages: (I) pre‐treatment, (II) permeation, (III) self‐assembly, and (IV) solvent replacement. First, the skin substrate (tanned leather) was prepared using a series of standard leather‐making techniques, such as fleshing, soaking, degreasing, deliming, unhairing, softening, and tanning. These procedures were created to optimize the retention of collagenous elements while effectively removing debris, hair, and non‐collagenous elements from the interior or exterior of the skin. At the same time, the collagen fiber skeleton with a 3D woven structure was obtained by splitting the skin to remove the surface layer (Step I). Then, after electrically conductive PMMA@MXene (PM) spheres were evenly distributed in polyvinyl alcohol (PVA)/gelatin (GEL)/carboxylated cellulose nanofibers (CNFs)/borax (named PGCB) solution, the obtained PGCB/PM pre‐gel solution was applied to collagen fiber skeleton. Utilizing the numerous binding sites and natural pore structure, PGCB/PM pre‐gel solution permeated uniformly and thoroughly in the hierarchical structure of the collagen fiber skeleton via vacuum filtration at room temperature (Step II). Subsequently, PGCB/PM pre‐gel solution, which was embedded in the skeleton of collagen fibers, underwent an in situ phase transition process at low temperature to form pre‐gel and fix the shape. Thanks to the interpenetrating network structure by interwoven pre‐gel and collagen fiber as well as the numerous hydrogen bonds between their active functional groups, PGCB/PM pre‐gel was firmly bonded to the collagen fiber skeleton (Step III). At last, PGCB/PM pre‐gel encapsulated collagen fiber structure was treated with a mixture of water, glycerin, and electrolyte NaCl through a solvent replacement process. The final IECS was obtained when PGCB/PM pre‐gel was transformed into PGCB/PM organohydrogel containing NaCl (PGCB/PM/N), which demonstrated excellent mechanical, conductive, sensing, anti‐freezing and moisture‐retention properties (Step IV).

Collagen fiber skeleton and PGCB/PM/N were primary components of IECS. The former served as a flexible substrate and the latter acted as conductive material, both of which worked cooperatively to improve the performance of final products. On the one hand, the structure of the collagen fiber skeleton was incredibly loose and ranged from nanoscale to macroscale. In the meantime, the abundance of pores and active groups that existed between collagen fiber skeleton offered the structural foundation for the fabrication of sensing material PGCB/PM/N. On the other hand, PVA and GEL, as flexible polymers, formed independent macroscopic structures and complete 3D skeletons between collagen fibers in the presence of cross‐linking and reinforcing agents. An interpenetrating network and a continuous conductive network entangled with collagen fiber bundles were established as a result of multiple hydrogen bonds between polymer chains and collagen fibers. In this process, the mutual cooperation of various components provided the possibility for the multifunctional integration of IECS. The details were as follows. (I) PVA with excellent mechanical properties was selected as the main network material. Meanwhile, GEL acted as the secondary network and interacted with PVA to produce interpenetrating polymer networks and increase flexibility. (II) CNFs, as reinforcing agents, interspersed between polymer chains under the action of hydrogen bonds, which are generated between the active groups on CNFs (hydroxyl and carboxyl) and polymer molecules (hydroxyl, carboxyl, amino, and amide). (III) Borax containing tetraborate ions was used as a cross‐linking agent to form dynamic borate ester bonds with the vicinal diol of PVA chains, further enhancing the mechanical properties and maintaining the integrity of network structures. At the same time, the migration of ionized Na^+^ provided ionic conductivity. (IV) Most notably, IECS utilized free ions (Na^+^, Cl^−^), and PMMA@MXene spheres as conductive materials. The former reached ionic conductivity by feasible transport channels with pore structure and water, while the latter accomplished electron transport by creating an intact conductive network. The combined impact of the two contributed to significant changes in ionic and electronic transport pathways under deformation, thus leading to dual conductive capability and super sensitivity to the strain of IECS. (V) By forming high‐density hydrogen bonds with water molecules, IECS based on the mixed solution of water and glycerin lowered the temperature at which ice crystals form and slowed the rate at which water molecules evaporate. The favorable environmental stability of IECS was guaranteed due to its anti‐freezing and moisture‐retention characteristics, which laid a foundation for its comprehensive application in harsh environments.

### 2.2 Microstructure Characterization of IECS

**Figure 2:**
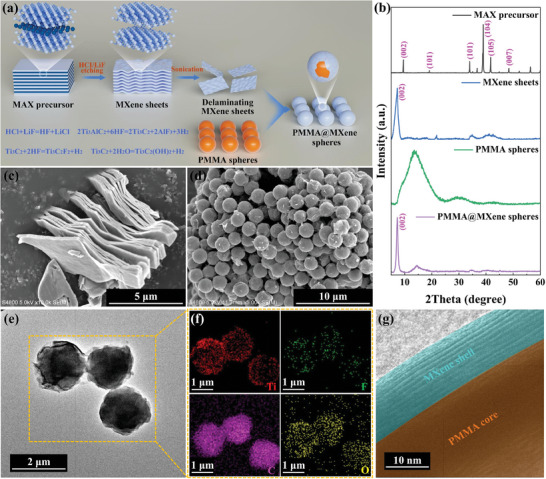
a) Schematic illustration of the preparation process of PMMA@MXene spheres. b) XRD patterns of MAX precursor, MXene sheets, PMMA spheres, and PMMA@MXene spheres. SEM images of c) MXene sheets and d) PMMA@MXene spheres. e) TEM image, f) element mappings, and g) HRTEM image of PMMA@MXene spheres.

As illustrated in **Figure** **2**, the morphologies of prepared MXene sheets and PMMA@MXene spheres were characterized to confirm the successful self‐assembly of MXene sheets and PMMA spheres. Figure 2a displayed the corresponding schematic illustration. The first step involved selectively etching of Al layer in MAX precursor by using HCl and LiF to generate MXene sheets with sufficient surface groups (such as hydroxyl, oxygen, and fluorine groups). The XRD patterns in Figure 2b allowed for the clear identification of the transition from MAX to MXene. The characteristic peaks at 34.0° disappeared corresponding to the crystalline planes (104) for MAX, while the characteristic peaks at 7.3° appeared corresponding to the crystalline planes (002) for MXene. Additionally, MXene was revealed to be typical 2D layered structures with thin and transparent layers by SEM and TEM images in Figure 2c and Figure S1a (Supporting Information). Based on SAED pattern in Figure S1b (Supporting Information), the typical hexagonal crystal structure of MXene sheets was identified. The obtained MXene sheets were then ultrasonically stripped for subsequent operations. Next, PMMA spheres and delaminating MXene sheets were used as cores and shells to create PMMA@MXene spheres by template method. Delaminating MXene sheets were forced to be tightly encapsulated on the surface of PMMA spheres through hydrogen bonding. Figure S2 (Supporting Information) illustrated that PMMA spheres had an average diameter of 2.01 µm. As a result of the ample coating of delaminating MXene sheets on PMMA spheres, the prepared PMMA@MXene spheres in Figure 2d had slightly larger average diameters of 2.03–2.07 µm and rougher surfaces. The TEM image, corresponding element mappings, and HRTEM image in Figure 2e–g further revealed that the perfect core@shell structure formed by tightly encasing multiple layers of delaminating MXene sheets surrounding the surface of PMMA spheres. F and Ti elements primarily originated from delaminating MXene sheets, whereas C and O elements were found in both delaminating MXene sheets and PMMA spheres. It was evident that the original spherical structure of PMMA templates was perfectly preserved and the delaminating MXene sheets were equally distributed on their surfaces. The XRD pattern of PMMA@MXene spheres in Figure 2b also demonstrated the simultaneous existence of characteristic peaks belonging to MXene sheets and PMMA spheres, which further confirmed the success of their recombination.

**Figure 3:**
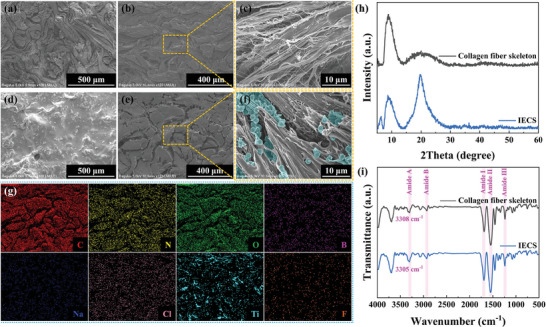
SEM images of a–c) collagen fiber skeleton and d–f) IECS: (a, d) surface, (b, e) cross‐section, and c, f) partially enlarged images. g) Element mappings of IECS corresponding to **Figure** **3**e. h) XRD patterns and i) FT‐IR spectra of collagen fiber skeleton and IECS.

PGCB/PM/N assisted in the assembly of PMMA@MXene spheres into IECS. Notably, the successful construction of IECS was made feasible by temperature‐induced sol‐gel transition characteristics, as shown in Figure S3 (Supporting Information). PGCB/PM pre‐gel solution was discovered to be coated at room temperature on the surface of numerous substrates with arbitrary shapes. Afterward, the temperature was adjusted to achieve phase transformation and generate the composites of PGCB/PM pre‐gel and substrates. Also, the subsequent solvent displacement process did not change their macroscopic structure. Therefore, the above established a foundation for the construction of IECS. The microscopic morphology and bonding structure of IECS were studied to clarify its hierarchical structure and formation mechanism. The collagen fiber skeleton was well preserved despite PGCB/PM/N filling up the interstitial spaces between fiber structures, according to SEM images in Figure 3a–f. Concurrently, the spherical structure of PMMA@MXene spheres was observed on the surface of collagen fiber bundles. Furthermore, the distribution of different elements on the cross‐section of IECS in Figure 3g demonstrated the uniform penetration and assembly of PGCB/PM/N within the collagen fiber skeleton, which set the platform for the formation of the dual conductive path and interpenetrating network structure in IECS.

As demonstrated in Figure 3h,i, the chemical interaction in IECS was further investigated by employing XRD and FT‐IR. In Figure 3h, the collagen fiber skeleton had two distinct diffraction peaks at 8.8° and 20.1°. The former, which came from the comparatively regular structure of collagen fibers, meant the distance between molecular chains of collagen fibers. The latter represented the diffuse reflection of hierarchical structure, resulting from the structure of amorphous regions in collagen fibers. When combined with PGCB/PM/N, the diffraction peak for IECS at 20.1° became stronger, while the diffraction peak at 8.8° weakened and shifted to a lower angle direction. These disclosed that the filled PGCB/PM/N increased the distance between molecular chains of collagen fibers and enhanced the amorphous structure of collagen by destroying the original van der Waals forces and hydrogen bonds between collagen fibers.^[^ ^25^ ^]^ At the same time, there was no diffraction peak of titanium oxide in IECS, which indicated that PMMA@MXene spheres did not oxidize during the preparation of IECS. This result was also illustrated by the basically constant conductivity of PMMA@MXene spheres before and after treatment in Figure S4 (Supporting Information), and the corresponding treatment process was detailed in Section 4.4 (Fabrication of IECS). Furthermore, Figure 3i showed that the collagen fiber skeleton and IECS had comparable positions of characteristic absorption peaks in amide I, II, and III bands, demonstrating that the introduction of PGCB/PM/N did not change the three‐stranded helical structure of collagen fibers. Also, the typical absorption peak of IECS in the amide A band was located at 3305 cm^−1^. It was shifted toward a lower wavenumber compared with the collagen fiber skeleton, suggesting the generation of hydrogen bonds between collagen fibers and polymer networks.^[^ ^25^ ^]^ To clarify, the original hierarchical structure of the collagen fiber skeleton was not altered after the functional modification with PGCB/PM/N. This made IECS exhibit similar characteristics to natural collagen fiber skeleton.

### 2.3 Mechanical and Conductive Properties of IECS

A number of samples, including S, PS, ICS, and ECS, were used as comparison models in order to better understand the effect of each component on the overall performance of IECS. Where, S, PS, ICS, and ECS represented the collagen fiber skeleton, collagen fiber skeleton combing with PGCB, ion‐conducting collagen fiber‐based e‐skin, and electron‐conducting collagen fiber‐based e‐skin, respectively. And these samples are listed in Table S2 (Supporting Information).

**Figure 4:**
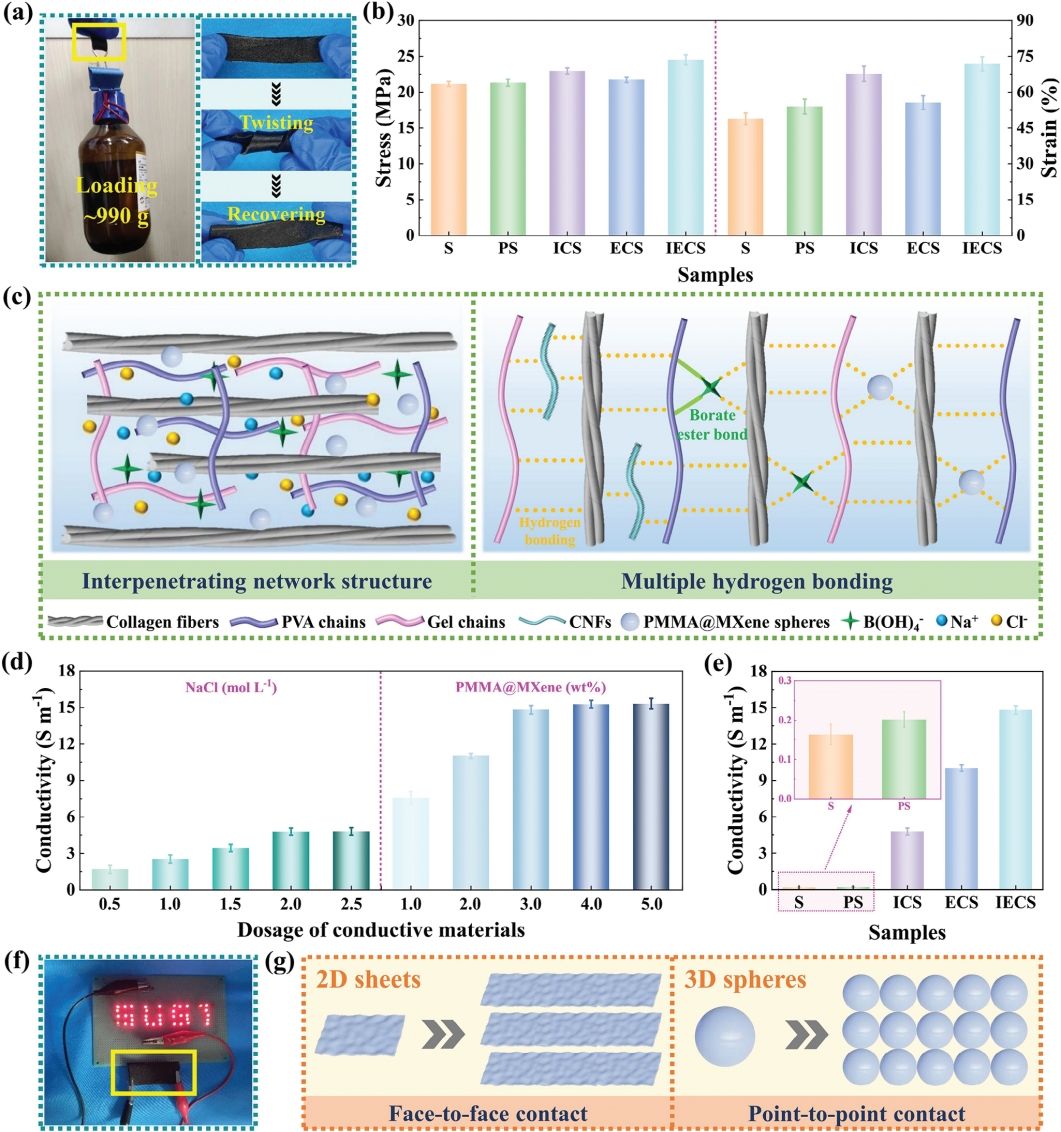
a) Visualized photos of IECS treated by manually hanging of ≈990 g weight and twisting. b) Mechanical properties of S, PS, ICS, ECS, and IECS. c) Schematic illustration of interpenetrating network structure and multiple hydrogen bonding of IECS. d) Conductivity of IECS prepared with different dosages of conductive materials. e) Conductive performances of S, PS, ICS, ECS, and IECS. f) Visualized photos of IECS as part of a circuit making LEDs glow. g) Schematic illustration of different conductive mechanisms based on face‐to‐face and point‐to‐point contacts.

The stability and durability of IECS were given support by its mechanical characteristics. IECS was particularly flexible and deformable, as demonstrated in **Figure** **4**a, enabling mechanical manipulation such as repeated twisting. The stress and strain of S, PS, ICS, ECS, and IECS were further measured to demonstrate their mechanical properties in Figure 4b. It was discovered that IECS possessed better mechanical properties than the other four samples, which was attributed to the contributions of network structure and conductive materials in Figure 4c. First, the formation of interpenetrating network structure was one of the reasons for the enhanced mechanical property of PS compared to S. The uniform permeation and dynamic cross‐linking of organohydrogel in the interstitial space of collagen fiber skeleton made them interpenetrate each other, thus forming interpenetrating network structure. The deformation process of the interpenetrating network involved greater energy dissipation. This was the reason why PS with an interpenetrating network structure had superior mechanical properties compared to S with a single network structure. Meanwhile, multiple hydrogen bonding between the collagen fiber skeleton and organohydrogel was produced with the assistance of hydroxyl, carboxyl, amino, amide, and other active groups, which was also valuable for the energy dissipation of PS. Second, the mechanical properties of ICS and ECS were further improved by the addition of NaCl and PMMA@MXene spheres, respectively. On the one hand, the process of salting‐out, symbolized by NaCl, boosted macromolecular aggregation and interaction by reducing electrostatic repulsion between polymer chains. The resulting large number of junctions prompted more cross‐linking of polymer chains, which generated porous 3D structures and dense physical cross‐linked networks. Furthermore, Na^+^ and Cl^−^ ions also possessed a tendency to immobilize polymer chains because of dipole interactions, triggering the development of positively and negatively charged chain segments. The improvement in the mechanical properties of ICS compared to PS was the consequence of physical cross‐linking generated by the entanglement of these segments. On the other hand, PMMA@MXene spheres formed multiple hydrogen bonds with polymer network and collagen fiber skeleton with the assistance of hydroxyl, oxygen, and fluorine groups, as well as borate ester bonds with PVA molecular chains by means of tetraborate ions. The rigidity of PMMA@MXene spheres and the formed dynamic sacrifice bonds enabled the polymer network to evenly distribute stress during deformation, promoting the energy dissipation process. So the mechanical properties of ECS were superior to PS. Finally, IECS featuring interpenetrating network structure and dual conductive paths was constructed upon these foundations. The resulting stress and strain of IECS were 24.51 MPa and 71.82%, respectively, proving significant improvement in mechanical properties attributed to the synergistic effect of each component. Notably, the developed IECS had higher mechanical strength and lower stretchability than PGCB/PM/N without a collagen fiber skeleton in Figure S4 (Supporting Information). This was because the 3D woven structure of the collagen fiber skeleton carried and transferred stress more effectively. As a result, IECS based on collagen fiber skeleton was difficult to collapse and exhibited excellent mechanical strength. In terms of strain, the stretchability of the collagen fiber skeleton was limited by the tightly woven 3D structure. As soft materials, PGCB/PM/N around collagen fiber bundles improved the deformation ability of IECS to a certain extent. Thus, it was better than the original collagen fiber skeleton but was still inferior to PGCB/PM/N.

In addition to mechanical properties, good electrical conductivity was also crucial for IECS applications in the field of soft sensing, which was closely related to the dosage of conductive materials. From Figure 4d, it was found that the conductivity of IECS increased from 1.68 to 4.81 S m^−1^ with the increase of NaCl content. The reason for this was that the ion movement in IECS was enhanced and more ion migration channels were provided. Subsequently, the changes in conductivity tended to level off due to the restriction of the dense polymer network triggered by salting out. By changing the amount of PMMA@MXene spheres, the conductivity of IECS was further adjusted to 15.31 S m^−1^. The reason was that the separated PMMA@MXene spheres gradually connected with each other and formed a complete electronic conductive path with the increase of PMMA@MXene sphere's dosage. However, the excess PMMA@MXene spheres made no contribution to the formation of a conductive pathway. After consideration, IECS with conductivity of 14.82 S m^−1^ was taken as the optimal sample for subsequent experiments, in which the dosages of NaCl and PMMA@MXene spheres were 2.0 mol L^−1^ and 3.0 wt.%, respectively. For comparison, the conductivity of S, PS, ICS, ECS, and IECS is shown in Figure 4e. The conductivity of S and PS was only 0.16 and 0.20 S m^−1^ in the absence of NaCl and PMMA@MXene spheres, respectively, which was due to the residual free ions of the tanning process and cross‐linking agent. With the addition of NaCl and PMMA@MXene spheres, the corresponding conductivity of ICS and ECS significantly increased to 4.79 and 10.02 S m^−1^, respectively, which was closely related to the high concentration of free ion migration and the complete physical contact of electron conductive network. On these bases, the conductivity of IECS with dual‐mode conduction induced by NaCl and PMMA@MXene spheres was increased to 14.82 S m^−1^. To further show its conductive capability, Figure 4f proves how IECS was utilized as a conductor to light LEDs.

Remarkably, the unique microstructures of PMMA@MXene spheres inspired additional research on the conductive characteristics of IECS. By using the sample prepared with traditional MXene sheets as the control object (referred to as IECS‐MS), the relationship between the morphology of the electrically conductive network and the performance of the corresponding e‐skin was further clarified. As an electrically conductive network was essentially formed, the amount of PMMA@MXene spheres and traditional MXene sheets was 3.0 and 1.0 wt.%, respectively, according to the results in Figure 4d and Figure S5 (Supporting Information). For IECS‐MS, traditional MXene sheets were presented in a 2D form and electrically contacted by face‐to‐face stacking,^[^ ^26^ ^]^ as illustrated in Figure 4g. For IECS, the mass ratio of PMMA spheres to MXene sheets was 10:1 during the preparation process of PMMA@MXene spheres, meaning that 3.0 wt.% of PMMA@MXene spheres was made up of roughly 2.73 wt.% of PMMA spheres and 0.27 wt% of MXene sheets. Compared to 1.0 wt.% of traditional MXene sheets, the actual amount of conductive materials in PMMA@MXene spheres was only ≈0.27 wt.% when an electrically conductive network was essentially formed. It demonstrated that PMMA spheres served as essential templates for isolating structures during the development of electrically conductive networks. The interaction between PMMA@MXene spheres was point‐to‐point contact, as shown in Figure 4g, which made it easier to produce a 3D conductive network and lower percolation threshold. Furthermore, in terms of sensing, PMMA@MXene spheres with point‐to‐point mode were more likely to separate under tensile strain,^[^ ^27^ ^]^ resulting in a more pronounced change in the relative resistance of IECS. In addition, 0D materials such as silver nanoparticles (AgNPs) also had excellent electrical conductivity. Their disordered migration under external forces was conducive to the destruction and reconstruction of conductive networks, resulting in significant changes in the resistance of the sensor. However, the low aspect ratio of 0D materials made the threshold of conductive percolation usually high, which meant that a complete conductive network could only be formed in the polymer at a larger dosage. It was not conducive to the improvement of mechanical properties and the reduction of costs of the sensor. Therefore, PMMA@MXene spheres with spherical structures were more conducive to the improved overall performance of IECS.

### 2.4 Electronic Behavior and Sensing Mechanism of IECS

Strain sensing behavior is an inherent property of smart materials, which makes them possible as strain sensors.^[^ ^28^, ^29^ ^]^ Various theoretical models have been developed to explain the mechanisms of self‐perception.^[^ ^30^, ^31^, ^32^ ^]^ In general, the shape of conductive networks and the distance between conductive materials affect ion migrations and electronic transitions, resulting in changes in the output electrical signal of smart materials.^[^ ^33^ ^]^ The above analysis showed that the dual conductive network proposed in this work formed a large number of conductive pathways in IECS. Meanwhile, the presence of NaCl‐induced free ions and PMMA@MXene spheres‐induced conductive sphere arrays provided different sources for signal variation. Therefore, the unique conductive network structures had a significant impact on the strain‐sensing behavior of IECS.

**Figure 5:**
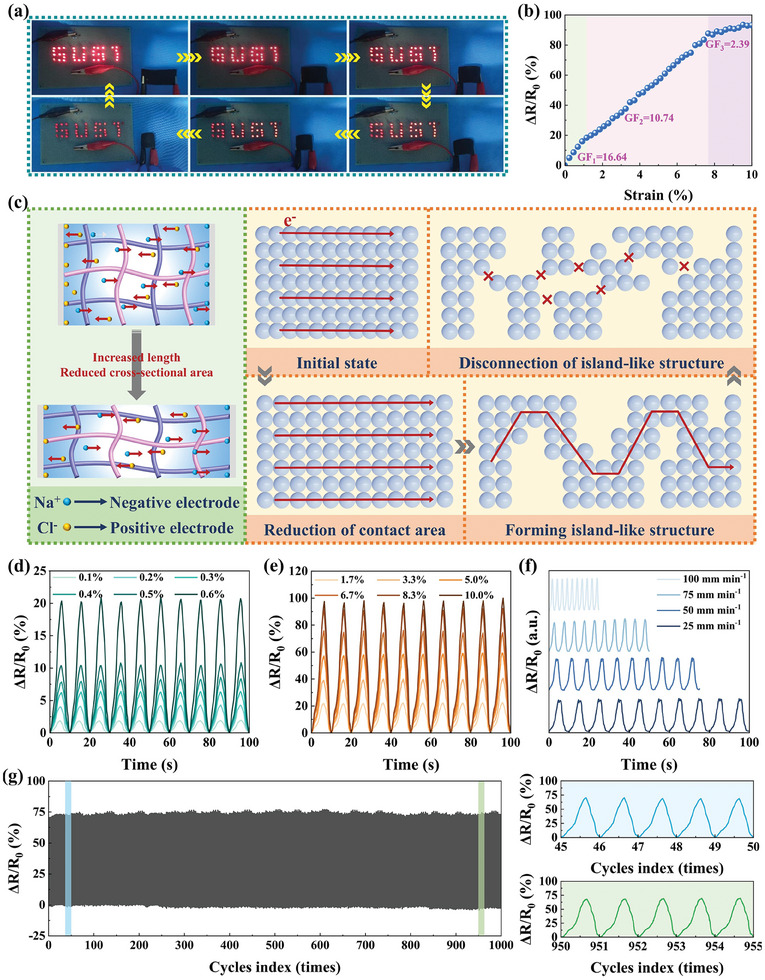
a) Change in LEDs brightness with the strain of IECS connected in the electric circuit. b) Relative resistance variation of IECS at different strains. c) Schematic illustration of the strain deformation of IECS. d–f) Relative resistance variation of IECS at small strains (<1%), large strains (1–10%), and strain frequencies. g) Relative resistance change of IECS for 1000 loading‐unloading cycles at 6.7% strain.

As a conductor, IECS was connected to an electric circuit. And the performance of IECS in sensing for bending strain was visually evaluated by observing the change of LEDs brightness with bending angle. As illustrated in **Figure** **5**a, the brightness of LEDs connected to an electric circuit gradually decreased with an increase in bending angle. Then, LEDs instantly brightened as the bending strain decreased, which signified a quick resistance response of IECS to bending strain. Importantly, it was intuitively observed that IECS still had good conductivity during the entire bending process, indicating that the conductive network of IECS was stable even under a large bending angle. Sensitivity (GF), one of the most important parameters to evaluate sensing performance, was assessed by displaying relative resistance change (ΔR/R_0_) under bending. This was determined using the formula of GF = (ΔR/R_0_)/ɛ. Where ΔR represented the resistance change before and after bending. R_0_ represented the resistance without bending and ɛ represented the applied strain to IECS. The relationship between bending angle and strain was defined and quantified in terms of ε = ±h/2r and c = 2rsin(l/2r). Of them, the thickness (h), arc length (l), chord length (c), and radius of curvature (r) of IECS were associated with bending strain. Figure 5b illustrates the ΔR/R_0_ curve of IECS at different bending strains. The ΔR/R_0_ value of IECS increased with the increase of bending strain. This was because the deformation of IECS resulted in increased length and decreased cross‐sectional area, which hindered the migration process of ions and electrons and significantly increased its resistance. Furthermore, the sensing process of IECS was fitted and divided into three stages. When the bending strain of IECS was 0–1.0, 1.0%–7.5%, and 7.5–10.0%, the corresponding GF was 16.64, 10.74, and 2.39, respectively, demonstrating exceptional sensitivity to bending strain under the synergistic action of the dual conductive path and collagen fiber skeleton. For comparison, the ΔR/R_0_ curves of ICS, ECS, and IECS‐MS at various bending strains were measured and presented in Figure S6 (Supporting Information). Significant differences were observed between different samples, illustrating the function of dual conductive paths and the construction of strain‐sensitive conductive networks derived from PMMA@MXene spheres in IECS. These were the main reasons affecting the change of conductive network structure and the sensing behavior of IECS under bending strain in terms of conductive materials. In addition, PGCB/PM/N and the sample of PGCB/PM/N formed only on the surface of the collagen fiber skeleton (denoted as IECS‐S) were subjected to the same sensing performance tests in Figure S7 (Supporting Information). As the results, both PGCB/PM/N and IECS‐S had lower ΔR/R_0_ values than that of IECS under the same bending strain, which suggested the importance of the interpenetrating network structure between collagen fiber skeleton and PGCB/PM/N. The change in network structure and the process of signal transmission were facilitated by the sliding and separation of collagen fibers. Therefore, the distinct 3D network and hierarchical structure of the collagen fiber skeleton, as well as the interpenetrating network structure between the collagen fiber skeleton and PGCB/PM/N, were helpful in augmenting the structural alterations of the conductive network in IECS.

Based on the above analysis and results, the sensing mechanism of IECS was proposed in Figure 5c. The dual conductive networks of ions and electrons dominated the strain response behavior of IECS. The former was directly correlated with the degree of deformation, whereas the latter relied on the actual physical contact of PMMA@MXene spheres. Regarding ion‐induced sensing behavior, the electric field of the initial state caused Na^+^ and Cl^−^ in IECS to move toward negative and positive poles, respectively. During this period, the resistance of IECS was associated with its inherent characteristics, specifically the internal resistance of ionic movement, which has been explained in detail in our previous work.^[^ ^34^ ^]^ The longer ion migration path and the narrower ion transport channel allowed IECS to have greater resistance as it strained, thus converting mechanical deformation into an electrical signal. To achieve electron‐induced sensing behavior, the 3D conductive pathway of IECS was created by connecting MXene sheets on the surface of PMMA@MXene spheres to one another. When no strain was applied, PMMA@MXene spheres in IECS were tightly assembled without cracks, and the inside electrons easily passed through. During the stretching process, the cracks usually developed and spread in the stress‐concentrated region of rigid PMMA@MXene spheres because PMMA@MXene spheres had higher Young's modulus and smaller elongation at break than collagen fiber skeleton and elastic polymer network. At this point, the resistance change was directly correlated with the formation, propagation, and disconnection of cracks between PMMA@MXene spheres.^[^ ^35^ ^]^ A tiny strain on IECS triggered overlapping PMMA@MXene spheres to slide and eventually separate, creating a few small fractures that ran perpendicular to the direction of strain. In the meantime, PMMA@MXene spheres started to aggregate together due to the cracks, and the interconnected island‐like structures that sustained partial electron transport were formed. During this time, the contact area between PMMA@MXene spheres decreased, resulting in the narrowing of the conductive channel and the increase of resistance in IECS (Stage I). As strain increased, the cracks gradually widened and mainly concentrated in the region of the gap. When the island‐like PMMA@MXene spheres split from each other, the formed bridge‐like structure further slowed the electron transport of IECS (Stage II). With a large increase of strain, PMMA@MXene spheres with bridge‐like structures elongated and fractured, producing multiple wide penetrating cracks. Due to the complete separation of some bridge‐like structures from each other, the conduction process of electrons was further restricted and the resistance of IECS was increased (Stage III). Finally, when all the connections between PMMA@MXene spheres were severed, the resistance of IECS reached infinity and the corresponding working range of the electron‐conducting network reached its limit. Therefore, IECS used for ultra‐sensitive strain detection in the whole range was feasible due to the mechanism of the formation, propagation, and disconnection of cracks between PMMA@MXene spheres.^[^ ^36^ ^]^

During the entire sensing procedure, the array structures formed by PMMA@MXene spheres concentrated external force on the contact points of the micro‐region in IECS, amplifying the structural alterations of an electron‐conducting network under strain. Meanwhile, the ability of IECS to detect strain was further enhanced by the hierarchical structure of collagen fibers and their interpenetrating network structure with polymer chains of PGCB/PM/N. In conclusion, IECS demonstrated a more significant ΔR/R_0_ value and superior strain sensing ability at all stages owing to the dual structural changes of conductive networks with both ions and electrons and the deformation amplification effect induced by collagen fiber skeleton. Thus, the strategic synergy of the compositional and structural design of IECS made it a very promising force‐sensing part of an excellent piezoresistive sensor.

Additionally, as demonstrated in Figure 5d–f, the ΔR/R_0_ curves of IECS displayed continuous and stable signals without any discernible increase or decrease under dynamic bending tests at various small angles, large angles, and bending frequencies. The nearly constant curves showed that IECS was suitable for low hysteresis and good signal output stability when detecting bending deformations, indicating that it was versatile and could be used for a wide range of tasks. The durability of IECS was also evaluated in Figure 5g for widespread and practical application. After 1000 bending cycles, the electrical signals of IECS showed good amplitude and waveform without fluctuation, displaying exceptional durability and reliability to guarantee its long‐term availability. Besides, considering batch stability, the ΔR/R_0_ curves of IECS from different batches were tested and shown in Figure S8 (Supporting Information). As a result, the corresponding ΔR/R_0_ curves exhibited stable signals with only slight changes, which proved good batch stability of IECS.

### 2.5 Freezing Tolerance and Moisture‐Retention of IECS

The key requirement for the practical use of IECS as a variety of sensor devices is good environmental stability, which includes anti‐freezing properties at low temperatures and long‐term moisture retention. Therefore, the existence state of water molecules in IECS was crucial, which was closely related to the water/glycerin binary solvent system, inorganic salt NaCl, and collagen fiber skeleton. Considering that the NaCl‐induced salting‐out effect was essential in sample preparation, its influence on performance has been discussed in our previous work.^[^ ^34^ ^]^ Herein, PGCB/PM/N and IECS‐H_2_O were used as control samples to demonstrate the role of the solvent system and collagen fiber skeleton, especially the latter.

**Figure 6:**
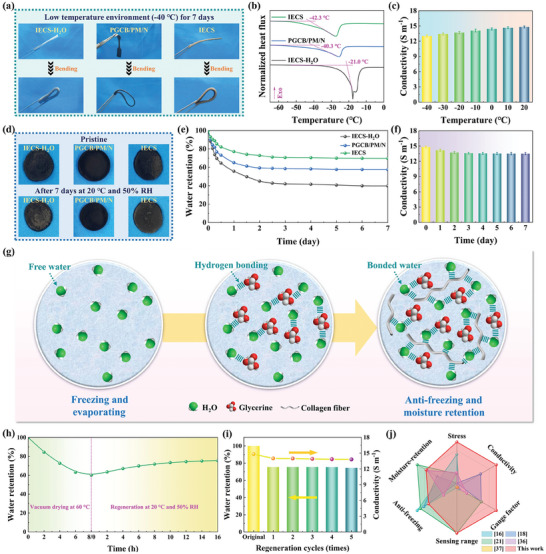
a) Visual comparison and b) DSC curves of IECS‐H_2_O, PGCB/PM/N and IECS. c) Conductivity of IECS at different temperatures. d) Visual comparison and e) moisture‐retention curves of IECS‐H_2_O, PGCB/PM/N, and IECS after placing for 7 days at 20 °C and 50% RH. f) Conductivity of IECS at different storage times. g) Schematic illustration of free water and bonded water of IECS. h) Moisture‐retention of IECS after vacuum drying at 60 °C for 8 h and regeneration at 20 °C and 50% RH for 16 h. i) Moisture‐retention and conductivity of IECS after 5 repeated drying and regeneration cycles. j) The comparison of comprehensive evaluation of IECS and other reported collagen fiber based e‐skins by conductive hydrogel.

To examine the anti‐freezing property of IECS at low temperatures, IECS‐H_2_O (without glycerin), PGCB/PM/N, and IECS were exposed to a low‐temperature environment (−40 °C) for 7 days. The flexibility of three samples was compared visually. IECS‐H_2_O hardened and froze, losing its initial flexibility. However, PGCB/PM/N and IECS were still able to withstand significant bending action, as seen in **Figure** **6**a. It was initially demonstrated that PGCB/PM/N and IECS performed well against freezing at low temperatures. In addition, DSC technology was used to confirm the freezing resistance of samples in Figure 6b. The freezing point of water molecules was significantly lowered by glycerin and collagen fiber skeleton, as demonstrated that IECS‐H_2_O, PGCB/PM/N, and IECS showed clear crystallization peaks at −21.0 °C, −40.3 °C and −42.8 °C, respectively. Figure 6g explained the reason for the anti‐freezing property of IECS. The hydroxyl group of glycerin and the hydroxyl, carboxyl, amino, and amide groups of collagen fibers formed high‐density hydrogen bonds with water molecules, thus preventing the formation of ice crystals. Despite the presence of collagen fibers in IECS, the freezing points of PCGB/PM/N and IECS showed minimal difference. This was because the physical structure and chemical composition of collagen fibers were three helices and collagen, respectively, and their molecules had strong interaction forces. In low low‐temperature environment, collagen fibers still showed high structural stability, so the flexibility of IECS might be affected rather than completely lost. In the meantime, it was clear from Figure 6c that the conductivity of IECS fluctuated only slightly over the temperature range of −40 °C to 20 °C, showing good temperature stability. These results made it possible for the application of IECS in low‐temperature environments.

By comparing the water evaporation of IECS‐H_2_O, PGCB/PM/N, and IECS under the same conditions, their moisture‐retention capacity were assessed. The moisture‐retention of IECS‐H_2_O, PGCB/PM/N, and IECS after 7 days of storage at 20 °C and 50% RH was 39.8%, 57.6% and 69.7%, respectively, as shown in Figure 6d,e. The reason for the enhanced moisture‐retention performance of IECS was similar to its anti‐freezing mechanism. The moisture‐retention additive glycerin and collagen fiber skeleton formed strong hydrogen bonds with water molecules, lowering the saturated vapor pressure of water, as shown in Figure 6g. After being stored for 7 days, IECS continued to exhibit a high conductivity of roughly 13.50 S m^−1^, which remained almost unchanged from its initial state in Figure 6f. Even with the addition of glycerin and collagen fiber skeleton, IECS might still dry out in harsh conditions. However, nearby water molecules could be effectively absorbed owing to the high hygroscopicity of glycerin. This led to the self‐regeneration capacity of IECS in Figure 6h. IECS underwent the pre‐treatment of vacuum drying for 8 h at 60 °C. And then, the dehydrated IECS regained 75.5% of its initial weight after 16 h at 20 °C and 50% RH. Furthermore, Figure 6i illustrated that IECS had good environmental adaptability even after 5 repeat cycles, demonstrating a similar self‐regeneration performance. The excellent anti‐freezing, moisture‐retention, and self‐regeneration capabilities of IECS were suitable for long‐term use in practical applications.

In addition, IECS was compared to the previously reported functional collagen fiber‐based e‐skin in terms of mechanical, conductive, moisture‐retention, anti‐freezing, and sensing performances to illustrate its potential advantages in future intelligent flexible sensors,^[^ ^10^, ^13^, ^16^, ^18^, ^21^, ^36^, ^37^, ^38^, ^39^, ^40^, ^41^, ^42^, ^43^, ^44^, ^45^ ^]^ as shown in Figure 6j and Table S1 (Supporting Information). It demonstrated that IECS offered an abundance of multifunctional features and smart sensing.

### 2.6 Real‐life Demonstration as Wearable Sensor of IECS

**Figure 7:**
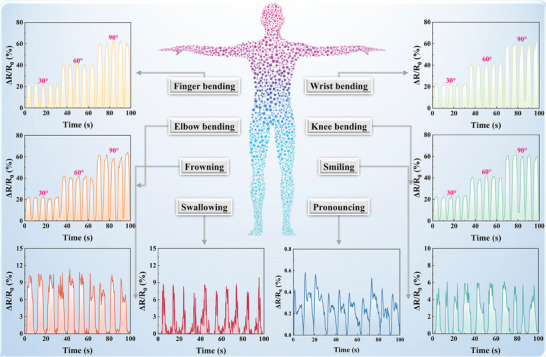
Real‐time monitoring of human movements by IECS and schematic diagram of sensing sites. Signals of relative resistance during vigorous activities (finger bending, wrist bending, elbow bending, and knee bending) and subtle motions (frowning, facial expressions such as smiling, swallowing, and speaking different phrases).

Based on the aforementioned characteristics, IECS was employed as a motion sensor for the detection of human physiological signals in **Figure** **7**. Strain‐dependent resistive signals were generated by IECS for movements, related to various body parts, including joint bending (fingers, wrists, elbows, knees, etc.), throat activity (pronouncing, swallowing, etc.), and facial expression (smiling, frowning, etc.). The obtained multiple signals showed consistency, reliability, and repeatability under various deformations, as evidenced by the somatosensory monitoring data collected from various body parts. In particular, IECS was attached to joints, such as the fingers, wrists, elbows, and knees, to record resistance signals and monitor joint movement. Tensile action initiated the ΔR/R_0_ value of IECS to increase when the joint of the human bent. Following the straightening of the joint, the ΔR/R_0_ value gradually reverted to its initial state. Furthermore, the ΔR/R_0_ value of IECS varied significantly with the bending angle of the joint. These phenomena were also observed in the resistance signals of other joint movements, which were explained by the piezoresistive effect. Under the action of applied strain, the physical deformation of IECS limited the transport path of ions and electrons, resulting in a change of resistance. Additionally, different response electrical signals were generated by IECS due to the movements of muscles and epidermis, when the throat was swallowing or pronouncing. The contraction and relaxation of facial muscles also elicited changes in response to electrical signals when the face displayed different expressions, such as frowning or smiling. Furthermore, substantial repeatability was indicated by the stable ΔR/R_0_ curves of IECS under continuous joint bending, throat activity, and facial expression. Therefore, IECS was an advantageous device for monitoring human movement in real‐time, and it created new possibilities for the applications of collagen fiber‐based e‐skin in artificial intelligence, wearable electronics, and human‐machine interfaces.

**Figure 8:**
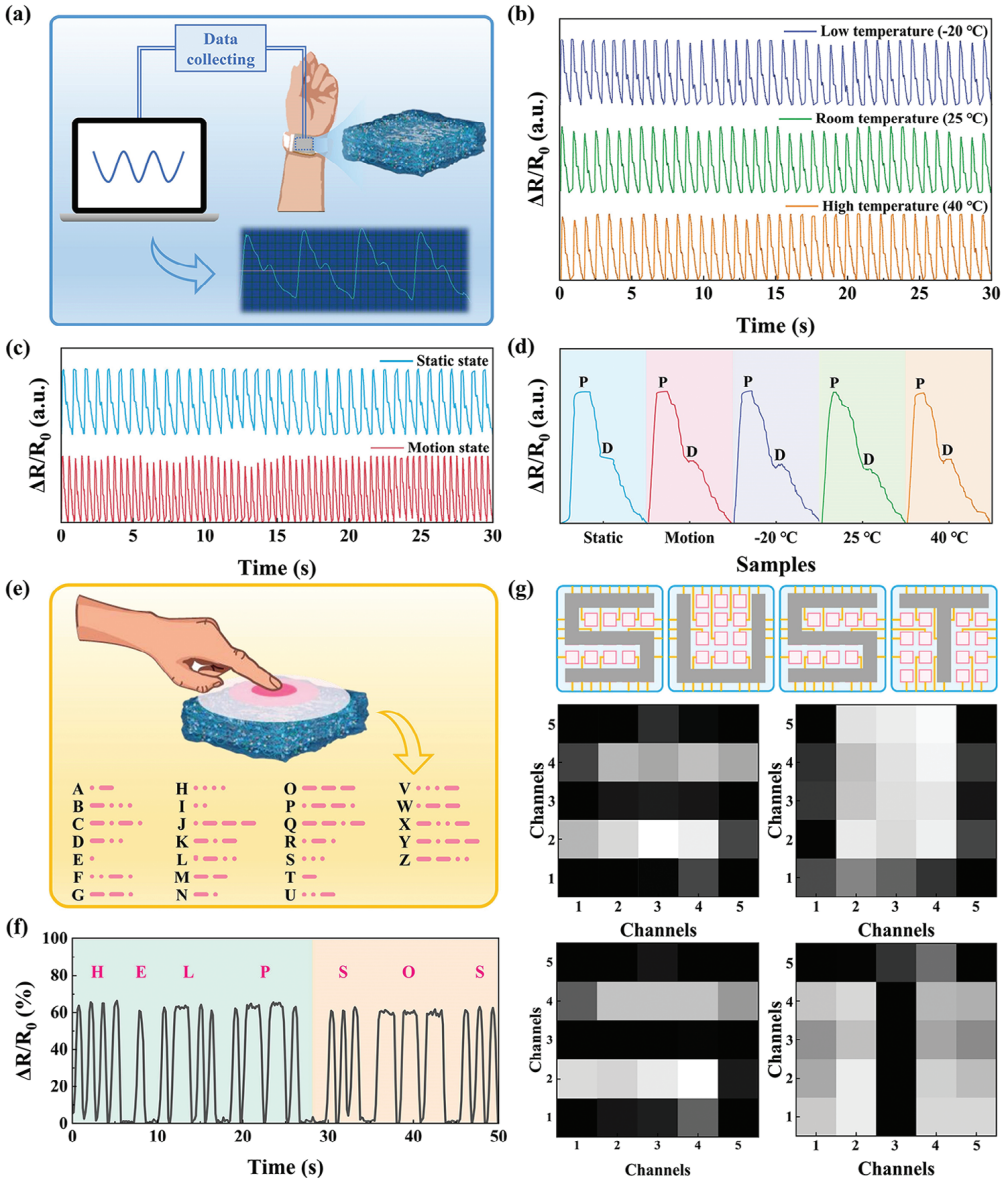
Schematic diagram of pulse pressure by IECS in real‐time monitoring. Signals of relative resistance under different b) temperatures (−20 °C, 25 °C, and 40 °C) and c) states (statics and motion). d) The corresponding detailed pulse waveform in **Figure** **8**b,c. e) Morse code table. f) Morse code to type “HELP” and “SOS” by finger pressure on IECS. g) Schematic diagram of 5 × 5‐pixel sensor array composed by IECS under the applied force of “S”, “U”, “S” and “T” and the corresponding signal distribution.

Importantly, the wrist pulse is the key physiological signal to determine arterial blood pressure and heart rate. IECS was integrated with signal processors, conversion circuits, communication circuits, and filters to monitor pulse signals by attaching them to the wrist of the volunteer in Figure 8a. When IECS was subjected to the pressure induced by pulse, its resistivity changed, which resulted in the change of output and the acquisition of signal. By placing the arm of the volunteer in a homemade device, pulse signals were recorded by IECS at different temperatures, as shown in Figure S10 (Supporting Information). Under different temperatures (−20 °C, 25 °C, and 40 °C) and states (statics and motion), Figure 8b–d showed a typical radial artery pulse waveform, where clearly distinguishable peaks and a late augmentation shoulder were observed in one cycle. Due to the excellent environmental stability of IECS, these data remained stable at the test temperatures. Moreover, the state of the human body was closely related to pulse rate. So the strength of motion state was judged from Figure 8c. These above results held the promise of IECS for more detailed health diagnoses. Additionally, Morse code was known to be an effective, real‐time, and time‐honored way of communication in coded languages. For coded languages, different combinations of “dots” and “dashes” represented different letters in the alphabet, as shown in Figure 8e. In this work, electrical signals caused by finger pressure were converted to Morse code. Specifically, sharp peaks produced by short‐term stressing were designated as the meaning “dots”, while peaks with the plateau produced by longer‐term stressing were defined as being in the “dashe” state. Therefore, IECS was employed to build a mechanoresponsive sensor for information transfer. According to the Morse code table, the combination of the letters “HELP” and “SOS” was successfully detected. To further explore the practical sensing applications of IECS, a fully integrated array of wearable sensors was assembled as artificial electronic skin and was applied to detect spatial pressure distribution. In this case, 25 IECS were assembled into a sensing array and interconnected by copper wires. Each IECS acted as a sensor unit. When external pressure was applied to the surface of the sensor unit, the contact position was recognized immediately and the corresponding electrical signal was detected. Thus, the pressure distribution in the space was recorded. As shown in Figure 8g, when the sensor unit was pressed under glyphs “S”, “U”, “S” and “T”, different electrical signals were output. The results showed that the flexible sensor array constructed by IECS had great potential for application in the field of e‐skin and human‐computer interaction.

## 3 Conclusion

In conclusion, the multifunctional natural skin served as inspiration for the design and construction of a temperature‐induced “penetration and self‐assembly” strategy, resulting in an IECS‐based collagen fiber skeleton with a variety of biological functions and smart sensing capabilities. From a structural point of view, PGCB/PM/N in collagen fiber skeleton encountered uniform penetration and dynamic cross‐linking, enabling the two to interpenetrate and form multiple hydrogen bonding interactions in IECS. It ensured that PGCB/PM/N appeared in the form of encased collagen fibers while maintaining the naturally 3D hierarchical structure of the collagen fiber skeleton. From a performance angle, the collagen fiber skeleton was loaded with conductive materials (NaCl, PMMA@MXene spheres), organohydrogel network (PGCB), and an antifreeze agent (glycerin). The resultant IECS simultaneously maintained a variety of biological functions, including flexibility, tensile strength (24.5 MPa), conductivity (14.82 S m^−1^), sensing ability (GF was 16.64, 10.74, and 2.39 in the bending strain of 0–1.0, 1.0%–7.5%, and 7.5–10.0%, respectively), freezing resistance (−42.8 °C) and moisture‐retention (the residual weight rate reached ≈69.7% after storage at 20 °C and 50% RH for 7 days). IECS with both ionic and electronic dual conductive networks achieved high sensitivity over a wide working range by utilizing the directional migration of free ions under large deformation and the structural change of electron conductive network under small external force. Significantly, the external force sensing capability of IECS was also improved by the crack creation and propagation caused by PMMA@MXene spheres and the deformation amplification effect caused by collagen fibers. Besides, the glycerin/water binary solvent system and collagen fiber skeleton interacted with water molecules to form high‐density hydrogen bonds, which was the reason for its enhanced environmental stability. Based on a variety of fascinating characteristics, natural skin‐derived IECS was perfect for feasible employs like monitoring human movement, pulse frequency, cipher transmission, and pressure distribution. Wearable electronics, artificial intelligence, and human‐machine interfaces were anticipated to reap benefits greatly from the true and adaptable e‐skin platform of IECS.

## 4 Experimental Section

### Preparation of MXene Sheets

MXene sheets were prepared according to the facile wet chemical method by Gogotsi.^[^ ^46^ ^]^ Typically, 1.6 g of LiF powder was dissolved in 20 mL of HCl and stirred for 30 min at room temperature. Then, 1.0 g of MAX powder was slowly added to the above solution under ice bath conditions. After continuously stirring for 48 h at 35 °C, the mixture system was washed repeatedly with deionized water for ≈10 cycles (5 min × 3500 rpm for each cycle), at which time its pH value was ≈6–7. Finally, MXene sheets were obtained by freeze‐drying the collected sediment.

### Preparation of PMMA@MXene Spheres

PMMA@MXene spheres with 3D structures were constructed via the template method. First, MXene sheets were sonicated in deionized water for 20 min and then centrifuged at 3500 rpm for 30 min. The obtained black colloidal supernatant was MXene dispersion, and its concentration was measured by the mass of the solid and the volume of dispersion. After that, MXene dispersion was directly mixed with PMMA spheres dispersion and stirred vigorously for 2 h. The mass ratio of MXene sheets and PMMA spheres was controlled at 1:10. After centrifugation at 3500 rpm for 5 min, PMMA@MXene spheres were obtained by freeze‐drying the black sediment.

### Pre‐treatment of Collagen Fiber Skeleton

The animal skin pieces were obtained from chrome‐tanned cattle hide, and then immersed in deionized water for 24 h to remove dirt and residual chemicals. During this period, deionized water was renewed every 6 h. Finally, the skin sample, denoted as collagen fiber skeleton, was freeze‐dried and stored at room temperature for subsequent experiments.

### Fabrication of IECS

IECS with dual conductive paths of ions and electrons was obtained through the “permeation and self‐assembly” strategy. In the first step, CNFs powder was added to deionized water and passed through a high‐pressure homogenizer (at 10 000 psi 3 times) to obtain homogeneous CNF dispersion (1.02 wt.%). For the second step, CNF suspension, PVA, GEL, and PMMA@MXene spheres were uniformly dispersed in deionized water and then stirred at 95 °C until the powder was completely dissolved. Subsequently, borax solution was added by dripping slowly and stirring for another 0.5 h to obtain PGCB/PM pre‐gel solution. This process was carried out in Ar_2_ atmosphere to reduce the degree of oxidation of PMMA@MXene spheres. After that, the mixture was transferred onto the surface of the collagen fiber skeleton to permeate evenly by vacuum filtration under 0.098 MPa for 30 min. It was then placed in a freezer (−20 °C) for 12 h and thawed at ambient temperature for 2 h. After freeze‐thaw cycles, the composite of PGCB/PM pre‐gel and collagen fiber skeleton was immersed in the mixed solution containing NaCl, glycerin, and deionized water for 12 h to obtain IECS with PGCB/PM/N organohydrogel as sensing materials. In addition, S, PS, ICS, ECS, and PGCB/PM/N were also prepared for comparison, with detailed compositions listed in Table S2 (Supporting Information).

To evaluate the effect of oxidation on the electrical conductivity, especially in the second step, PMMA@MXene spheres were uniformly dispersed in deionized water without any other components. The obtained dispersion was placed in Ar_2_ atmosphere at 95 °C for the same time, and then the treated PMMA@MXene spheres were obtained by centrifugation and freeze‐drying. Finally, the conductivity of PMMA@MXene spheres before and after treatment was measured and compared.

In order to study its electrical behavior and sensing property, IECS‐MS was obtained by using traditional MXene sheets as electrically conductive materials rather than PMMA@MXene spheres. Meanwhile, IECS‐S was the sample that PGCB/PM/N only formed on the surface of the collagen fiber skeleton.

For low‐temperature tolerance and moisture‐retention performance, IECS‐H_2_O (without glycerin) was prepared by changing the types of solvent and other operations remained the same.

### Fabrication and Test of IECS‐Based Sensor

The obtained IECS were cut into strip‐shaped specimens with dimensions of 3 × 0.8 cm. Afterward, 2 pieces of copper foil connected with the metal wire firmly adhered to the two ends of the IECS specimen to assemble into the flexible sensor. Then, the VHB tape was used to encapsulate the based sensor to minimize ambient interference, as shown in Figure S11 (Supporting Information). For detecting human motion, the IECS‐based sensor was directly attached to the different joints of people, and its sensing performance was tested using an electrochemical workstation. This experiment was completed with the assistance of volunteers and informed written consent was obtained for publishing.

## Conflict of Interest

The authors declare no conflict of interest.

## Supporting information



Supporting Information

## Data Availability

Author elects to not share data.
