# Supplementary material for: Organohydrogel Based Electronic Skin Reinforced by Dual‐Mode Conduction and Hierarchical Collagen Fibers Skeleton
Source: Adv Sci (Weinh). 2024 Dec 16;12(6):2412934. doi: 10.1002/advs.202412934 (PMC11809366; doi:10.1002/advs.202412934)
Supplement: Supplementary file 1 — Supporting Information [file ADVS-12-2412934-s001.docx]

Supporting Information

**Organohydrogel Based Electronic Skin Reinforced by Dual-Mode Conduction and Hierarchical Collagen Fibers Skeleton**

*Ruyue Guo, Yan Bao*,* *Xi Zheng, Jie Chen, Wenbo Zhang, Chao Liu, and Jianzhong Ma**

R. Guo, Y. Bao, X. Zheng, J. Chen, W. Zhang, C. Liu, J. Ma

College of Bioresources Chemical and Materials Engineering, Shaanxi University of Science and Technology, Xi'an, 710021, PR, China.

1. mail: baoyan@sust.edu.cn; majz@sust.edu.cn

**Experimental Section**

*Materials:* Polyvinyl alcohol (PVA, polymerization degree 1799, 98~99% alcoholysis) and gelatin (GEL, ~250 g Bloom) were purchased from Shanghai Aladdin Reagent Co., Ltd. Carboxylated cellulose nanofibers (CNFs) with diameter of 50 nm and length of 1~3 μm, borax (Na_2_B_4_O_7_·10H_2_O), glycerine (C_3_H_8_O_3_) and lithium fluoride (LiF) were bought from Shanghai Maclin Biochemical Technology Co., Ltd. Sodium chloride (NaCl), polymethyl methacrylate spheres (PMMA), titanium carbide aluminum (Ti_3_AlC_2_, MAX, 300 mesh) and hydrochloric acid (HCl) were received from Tianjin Kemeiou Chemical Reagent Co., Ltd., Shanghai Plastic Fuju New Materials Co., Ltd., Tianjin Tianli Chemical Co. Ltd. and Laizhou Kailene Ceramic Materials Co., Ltd., respectively. Chrome-tanned cattle hide was supplied by Dahuanjiu Polygrace Leather Co., Ltd. All reagents used in the experiments were of analytical grade and received without further purification.

*Characterization:* The morphology and structure of samples were observed via Scanning Electron Microscopy (SEM, S4800, Japan) equipped with an Energy Dispersive Spectrometer (EDS) and Transmission Electron Microscopy (TEM, Hitachi, H-7650, Japan). X-ray Diffraction (XRD, D8 Advance, Germany) was used to determine the crystalline degree. Fourier Infrared Spectrometer (FT-IR, VECTOR-22, Germany) and Differential Scanning Calorimeter (DSC, 7020, Japan) was used to analyze the interactions of samples.

*Mechanical Property Measurement:* The mechanical properties of e-skins were tested on a tensile testing machine (Gotech Al-3000, China) in air environment. For tensile tests, the e-skins were made into dumbbell shapes with a length of 110 mm, a gauge length of 50 mm, a width of 10 mm. The stretch rate was fixed at 100 mm min^-1^ for the normal tensile tests. The ultimate stress was determined as the loading force divided by the original specimen cross-sectional area, and the ultimate strain was defined as the deformed length divided by the original length of the samples.

*Moisture Retention Measurement:* The moisture retention was tested by putting the e-skins into a container with constant temperature (20 °C) and humidity (50% RH) at varying times. The weight of e-skins during the test process was recorded at pre-set intervals, and the retained weight (wt%) was calculated.

*Anti-freezing Measurement:* Low temperature tolerance properties of e-skins were investigated using a differential scanning calorimeter (DSC, 7020, Japan) with NETZSCH intracooler. The e-skins were equilibrated at 0 °C and then cooled from 0 °C to -65 °C at a rate of 5 °C min^-1^. After the e-skins were kept at -65 °C for 10 min, the heating process was performed from -65 °C to initial 0 °C at a same rate of 5 °C min^-1^.

*Electrical Measurements:* The conductivity of e-skins was measured by alternating current impedance method using an electrochemical workstation (P4000+, the United States). During the test, a sinusoidal voltage amplitude of 0.01 V was applied and the frequency range was from 0.01 to 10^5^ Hz. The conductivity was determined according to Equation 1:

$\delta=d/{(R\times S)}$ (Equation 1)

where *d* represents the thickness, *R* means the resistance and *S* is the cross-sectional area of e-skins.

For the testing of sensing performances, the bending was carried out by a universal electromechanical tester, and the relative resistance variations (ΔR/R_0_) were recorded by the same electrochemical workstation simultaneously. The sensitivity of e-skins was evaluated by the gauge factor (GF), which is defined as Equation 2:

$GF=\frac{{(R-R_{0})}/{R_{0}}}{\varepsilon}=\frac{\Delta R}{R_{0}\times\varepsilon}$ (Equation 2)

where *R_0_* and *R* are the resistances of the original and stretched e-skins, respectively, *ε* is the applied strain to e-skins.

**Supporting Figures and Tables**


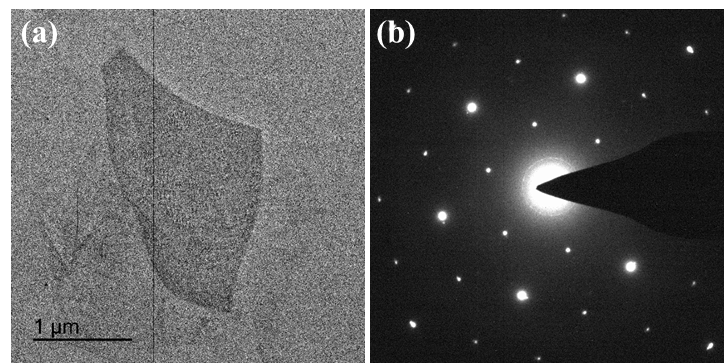


**Figure S1.** (a) TEM and (b) SAED images of MXene sheets.


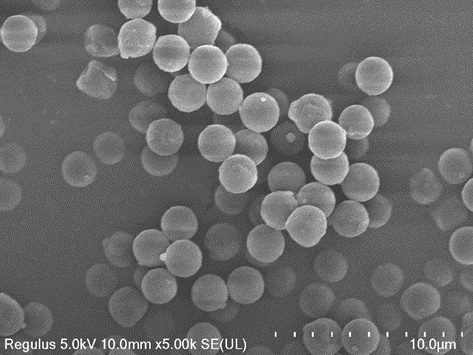


**Figure S2.** SEM image of PMMA spheres.


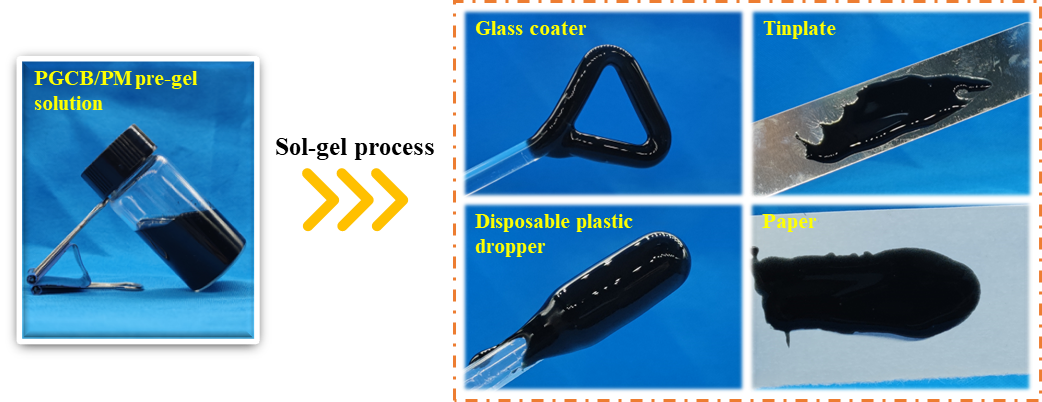


**Figure S3**. Appearance photos of the composite of different substrates with PGCB/PM pre-gel.

**Figure S4**. Conductivity of PMMA@MXene spheres before and after treatment.

**Figure S5**. Mechanical properties of PGCB/PM/N and IECS.

**Figure S6.** Conductivity of IECS-MS prepared with different dosages of traditional MXene sheets.


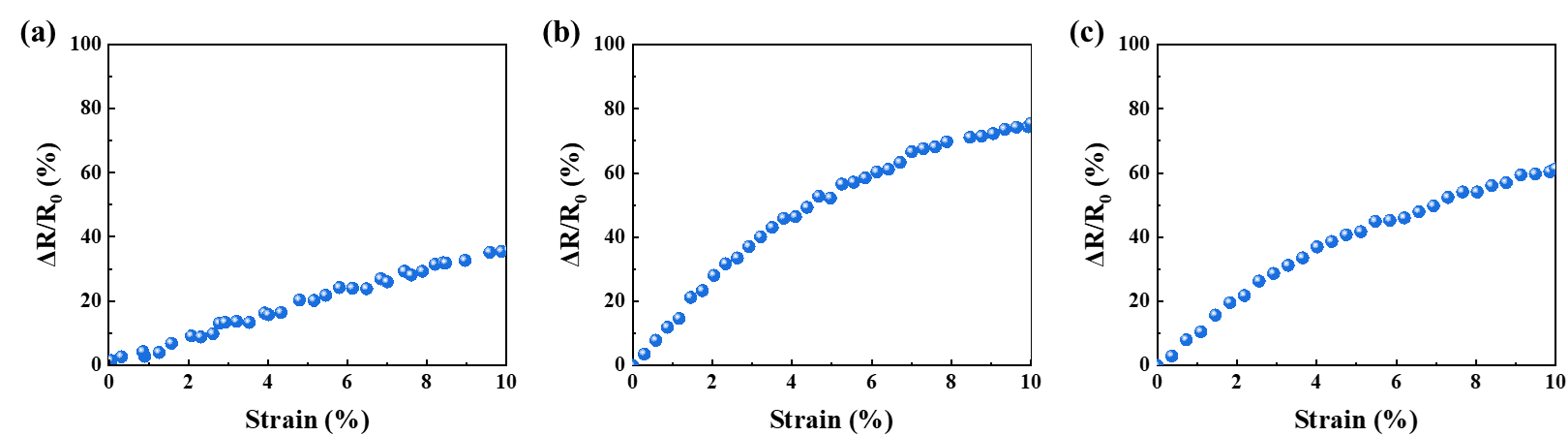


**Figure S7.** Relative resistance changes of (a) ICS, (b) ECS and (c) IECS-MS at different bending strain.


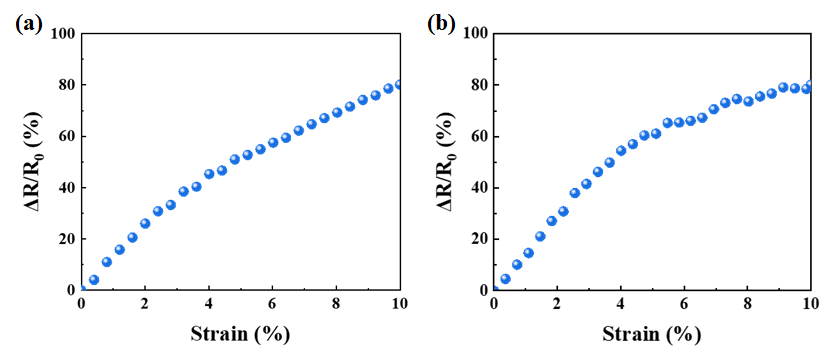


**Figure S8.** Relative resistance changes of (a) PGCB/PM/N, (b) IECS-S at different bending strain.

**Figure S9.** Relative resistance changes of IECS from different batches at 10% strain.

**Figure S10.** Schematic diagram of pulse pressure by IECS in real-time monitoring under (a) high temperature and (b) low temperature.

**Figure S11.** Schematic diagram of the fabrication of IECS based sensor.

**Table S1.** Comparison with typical collagen fiber based e-skin reported recently.

| **References** | **Sensing material** | **Stress** | **Conductivity** | **Gauge**  **factor** | **Freezing**  **tolerance** | **Moisture**  **retention** |
| --- | --- | --- | --- | --- | --- | --- |
| Our work | PMMA@MXene spheres and NaCl based organohydrogel | 24.51 MPa | 14.82 S m^-1^ | 16.64 | -42.8 ℃ | 69.7%  (20 ℃, 50% RH) |
| *Adv. Funct. Mater.*, 2023, 2304015. | AgNPs based organohydrogel | 2.4 MPa | 8.9 S m^-1^ | 10.3 | -50 ℃ | 45.0%  (25 ℃, 60% RH) |
| *Adv. Funct. Mater.*, 2023, 2212856. | AgNPs and NaCl based organohydrogel | 7.33 MPa | 1.03×10^-2^ S m^-1^ | 10.4 | -55 ℃ | 89.8  (25 ℃, 50% RH) |
| *Chem. Eng. J.*, 2023, 454, 140269. | Liquid metal based ionogel | 17.8 MPa | 0.25 S m^-1^ | 1.05 | -60 ℃ | — |
| *Nano Energy, 2023, 118: 108989.* | NaCl based organohydrogel | 8.14 MPa | 0.021 S m^-1^ | 4.21 | -20 ℃ | 67.0%  (25 ℃, 60% RH) |

| **References** | **Sensing material** | **Stress** | **Conductivity** | **Gauge**  **factor** | **Freezing**  **tolerance** | **Moisture**  **retention** |
| --- | --- | --- | --- | --- | --- | --- |
| *J. Mater. Chem. A, 2022, 10,*  *14555* | Multi-walled carbon nanotubes based organohydrogel | 0.25 MPa | 0.20 S m^-1^ | — | — | — |
| *Adv. Mater. Technol.*, 2019, 4, 1900442. | Carbon black | — | — | 21.49 | — | — |
| *Macromol. Rapid. Comm.*, 2022, 43, 2100873 | Poly(3,4-ethylenedioxythiophene): poly(4-styrenesulfonate) | — | — | 2326.84 | — | — |
| *Adv. Electron. Mater.*, 2020, 6, 2000549. | Carbon flake | — | — | 2009.5 | — | — |
| *Collagen and Leather*, 2023, 5, 25. | Multi-walled carbon nanotubes | — | — | 7238.92 | — | — |
| *J. Mater. Chem. A*, 2023, 11, 11773-11785. | Reduced graphene oxide | 6.2 MPa | 90 Ω sq^-1^ | — | — | — |

| **References** | **Sensing material** | **Stress** | **Conductivity** | **Gauge**  **factor** | **Freezing**  **tolerance** | **Moisture**  **retention** |
| --- | --- | --- | --- | --- | --- | --- |
| *ACS Appl. Mater. Inter.*, 2023, 15, 12350-12362. | AlCl_3_ | 0.15 MPa | 3.4 S m^-1^ | 13.65 | -60 ℃ | 80%  (25 ℃, 39% RH) |
| *Angew. Chem. Int. Ed.*, 2022, 61, 202200705. | Ag nanowires | 16.7 MPa | 0.8 Ω sq^-1^ | 0.81 | — | — |
| *J. Mater. Chem. C*, 2020, 8, 9748-9754. | Carbon black | — | — | 12.66 | — | — |
| *Chem. Eng. J.*, 2020, 392, 123672. | Polyaniline and multi-walled carbon nanotube | 9.2 MPa | 50 Ω sq^-1^ | — | — | — |
| *J. Mater. Chem. A*, 2023, 11, 726-741. | Polypyrrole and carbon black | 59.9 MPa | 6.5 S m^-1^ | 56.4 | — | — |

Note: — Means not mentioned.

**Table S2.** Composition of e-skins.

| **Samples** | **PVA** | **GEL** | **CNFs dispersion**  **(1.02 wt%)** | **PMMA@MXene spheres**  **(wt%)** | **Borax solution**  **(1 mg/mL)** | **NaCl**  **(M)** | **H_2_O:glycerine**  **(mass ratio)** |
| --- | --- | --- | --- | --- | --- | --- | --- |
| S | – | – | – | – | – | – | 8:2 |
| PS | 5.4 g | 0.6 g | 6.0 g | – | 1 mL | – | 8:2 |
| ICS | 5.4 g | 0.6 g | 6.0 g | – | 1 mL | 2.0 | 8:2 |
| ECS | 5.4 g | 0.6 g | 6.0 g | 3.0 | 1 mL | – | 8:2 |
| IECS | 5.4 g | 0.6 g | 6.0 g | 3.0 | 1 mL | 2.0 | 8:2 |

Note: — Means not added.
